# The Creation of the National Institute on Alcohol Abuse and Alcoholism: Responding to America’s Alcohol Problem

**Published:** 1995

**Authors:** Brenda G. Hewitt

**Affiliations:** Brenda G. Hewitt is special assistant to the director of the NIAAA, Bethesda, Maryland

**Keywords:** government agency, AOD abuse, AOD dependence, history, AOD-related (AODR) problems, legislation

## Abstract

Although they had founded their own national society and their own treatment program, people recovering from alcoholism in the 1960’s realized that Americans did not recognize the alcohol-dependent person’s plight. Thus, a few dedicated individuals set out to establish, through Federal legislation, a nationwide effort to combat alcoholism.

Twenty-five years ago, Congress passed and President Richard M. Nixon signed the Comprehensive Alcohol Abuse and Alcoholism Prevention, Treatment, and Rehabilitation Act of 1970 (Public Law [P.L.] 91–616). Referred to as the “Hughes Act” for the pivotal role played by Senator Harold E. Hughes in its passage, this law recognized alcohol abuse and alcoholism as major public health problems and created the National Institute on Alcohol Abuse and Alcoholism (NIAAA) to combat them. The road to the passage and signing of this legislation was not easy. In the end, it required the courage of a number of recovered alcoholics “going public,” the initiative and resourcefulness of a freshman U.S. Senator (who persevered despite a lack of funding for his Special Subcommittee on Alcoholism), and the intercession of three individuals in the waning hours of New Year’s Eve in 1970 to convince the President to sign P.L. 91–616 into law. The story of P.L. 91–616’s passage is remembered with pride by those who were there and is deserving of retelling on this occasion—the 25th anniversary of NIAAA—that is the legacy of their efforts to forever change the way Americans perceive and respond to alcohol problems.

## America’s Alcohol Problem

From the time the first colonists arrived in the New World, bringing their alcoholic beverages with them, Americans have had a problem with alcohol. That problem is a historic inability to reach any kind of a national consensus about the role of alcohol in American society. Americans’ inability to reach such a consensus, in turn, has led to fits and swings in public perception and, consequently, in public policy concerning how best to deal with the individuals whose use of alcohol causes difficulties and the difficulties these individuals cause. As observed by Selden E. Bacon, former director of the Center of Alcohol Studies at Rutgers University and eminent scholar on alcohol problems in America:

This complex set of problems over the past 150 years has been defined as a moral weakness problem and turned over to the churches, defined as an economic problem and turned over to market and price control authorities, defined as a youth learning problem and turned over to educators, defined as a crime problem and turned over to law enforcement and correction agencies (U.S. Congress 1970, p. 115).

Several major shifts have occurred in the way Americans have perceived and responded to alcohol problems from the colonial era to the temperance era (including Prohibition) and from the temperance and Prohibition era to the present (Moore and Gerstein 1981; Jung 1994).

### The Colonial Era: Alcoholism Is a Sin

During the colonial period in America, alcohol was very much a part of a community’s social life. Alcohol was used widely as both a beverage and a medicine, generally being considered a substance that was both enjoyable and healthful. Even drunkenness was tolerated so long as it did not interfere with a person’s livelihood or religious observance. In the colonial view, the problem was not alcohol, but the individual who used alcohol. Habitual drunkenness, which kept people from working and praying, represented a weakness of character and a sin against God and the church. Punishment was colonial America’s response to such weakness, and the stocks (i.e., structures that confined the arms and legs of social miscreants for public chastisement) were the colonial era’s equivalent of the alcoholism treatment facility.

### The Temperance Era: The Demon Is Rum

During the mid- to late-19th century, attempts to respond to alcohol problems shifted from trying to control the individual to trying to control the substance. With the Nation’s population transforming from an agrarian to an industrial society, new social problems, such as poverty and crime, began to emerge (Jung 1994). Each of these social ills was seen as connected to alcohol use. In response, a social reform movement was born that began to focus on eliminating alcohol use as a means of eliminating social problems. Aggressive public information and legislative activities of antialcohol groups, such as the American Temperance Society,^1^ the Women’s Christian Temperance Union, and the Anti-Saloon League, with their images of “demon rum” and ax-toting women, helped change Americans’ perceptions of alcohol problems and caused them, in response, to consider eliminating the substance. Moore and Gerstein (1981) note that during this period,

… the excessive drinker came to be seen as someone who was ravaged and transformed by an alien substance. Otherwise decent people could be transformed by drink to become dissolute, violent, or degenerate. Moreover, since alcohol was an addicting substance, even the most moderate drinker flirted with danger at the rim of every cup (p. 9).

Although alcohol-related health problems generally were not a major consideration during the temperance era, there is some historical evidence that even during the heyday of the antisaloon leagues, some attention was given to the social and health consequences of problem drinking. According to medical historian Phillip J. Pauly:

In the early 1890’s, Seth Low, a wealthy businessman, president of Columbia University and future mayor of New York [City], led the Sociology Group, an informal discussion circle of academic, commercial, and religious liberals interested in urban problems. In 1893 the group began to discuss alcohol, and became so persuaded of the need for knowledgeable, moderate action that they expanded to become a formal organization. The resulting Committee of Fifty for the Investigation of the Liquor Problem proposed to sponsor fact finding reports on the legal, economic, ethical, and … physiological aspects of alcohol use (Pauly 1990, pp. 366–392).

Not much came of the Committee’s efforts as the national climate moved toward Prohibition, and 1919 saw the passage of the Volstead Act, ushering in the legal abolition of alcohol consumption.

### Reaction and Inaction

Prohibition was both a success and a failure. According to the Cooperative Commission on the Study of Alcoholism,^2^ on the one hand:

… rates of problem drinking … decreased substantially during the early years of Prohibition. … reported deaths from liver cirrhosis also declined as did hospitalization for alcoholism. Arrests for public drunkenness were much lower than earlier (Plaut 1967, pp. 132–133).

On the other hand, “Prohibition was experienced as an intolerable abridgement of personal freedom by many Americans” (Plaut 1967, pp. 132–133). Thus, although Prohibition achieved the goal of reducing alcohol-related problems, Americans found the loss of personal autonomy in the matter of alcoholic beverages excessive and voted to repeal the Volstead Act in 1932.

The experience of Prohibition led next to an era, from the 1930’s through the 1960’s, in which alcohol-related problems generally were ignored. The Cooperative Commission aptly sums up the situation:

The unique place of alcohol beverages in American culture is evidenced by the fact that only one Amendment to the United States Constitution has ever been repealed; that was the Eighteenth, or Prohibition, Amendment. … The Prohibition Amendment was an attempt to “legislate morals”; repeal of the Amendment was taken as evidence that the American people felt this attempt had not succeeded, or indeed, was an example of the medicine’s being even worse than the illness. The hostile and apprehensive reaction to this particular means of regulation has unfortunately been transferred to the general idea of a comprehensive approach [to alcohol problems]. As a result, proposals to change drinking patterns—whether by educational, legislative, or other means—are still likely to evoke charge of disregarding the “lessons” of the Prohibition (Plaut 1967, pp. 14–15).

Fortunately, although many Americans tried very hard to forget about alcohol problems after Prohibition, changes were taking place in science and medicine, among public and private helping agencies, and, most importantly, among the group most affected by alcohol problems—the alcoholics themselves—to redefine alcohol-related problems as health problems.

## The Beginning of Change

The private and public sectors undertook actions that revitalized the national debate about alcohol-related problems and laid the groundwork for Federal legislation. In the private sector, both the founding in 1935 of the fellowship of Alcoholics Anonymous (AA) and the growing acceptance of alcohol abuse and alcoholism as health problems by scientific and medical organizations were pivotal events in helping to reintroduce the national policy debate on alcohol-related problems. The success of AA helped to demonstrate that alcoholics could recover. The establishment in the mid-1930’s of the Research Council on Problems of Alcohol at Yale University and the initial publication in 1940 of the scholarly journal *Quarterly Journal of Studies on Alcohol* were instrumental in recasting the public perception of alcohol abuse and alcoholism as problems that would yield to scientific solutions. The National Committee for Education on Alcohol (later called the National Council on Alcoholism), founded in 1944 by Marty Mann, the first woman to recover through AA, and researchers and physicians from Yale University, also helped to spread the word. By the 1950’s major health care organizations, such as the American Medical Association and the World Health Organization, began to address the health care aspects of alcoholism and the discrimination against alcoholics in health care settings. By the 1960’s these groups were joined by the American Psychiatric Association and the American Public Health Association in declaring alcoholism an illness (Plaut 1967). On the public side, by this time several States, such as California and Maryland, also had begun to develop programs to provide treatment and other supportive services to alcoholics, although these were often underfunded and not coordinated with the general health care system.

Despite these activities, public opinion was slow to move away from the view of alcohol abuse and alcoholism as moral or criminal issues. Federal programs to combat alcohol problems also were limited. By the 1960’s the National Institute of Mental Health (NIMH) in the U.S. Public Health Service had begun a very small program of grants in the alcohol area, leading to the establishment in 1965 of the National Center for the Prevention and Control of Alcohol Problems as a component of NIMH, with limited program authority and a limited budget. The situation with research was even more dismal. As asserted by the Cooperative Commission on the Study of Alcoholism in its 1967 report:

Additional information about the nature and causes of problem drinking is urgently needed. Past research in this area has been uneven and sporadic. … While special attention to alcohol problems is currently required … research in this field cannot be developed in isolation from investigations of a basic science nature and those on other medical and psycho-social problems (Plaut 1967, pp. 50 and 52).

There was a general feeling that the only way to sway public opinion and to address comprehensively alcohol abuse and alcoholism was from the national level through a highly placed and therefore highly visible Federal organization. For this, legislation was required, and it was to this end that the many disparate alcohol-related organizations came together in 1968.

One person who was involved intimately in the struggle for Federal recognition of and support for alcoholism legislation was the late Thomas P. Pike, a wealthy businessman from Los Angeles, CA. In his *Memoirs of Thomas P. Pike*, Pike (1979), himself a recovering alcoholic, speaks of the man whose name was to become indelibly linked to P.L. 91–616:

Then, in 1969, I met an extraordinary man in Washington [DC] who convinced me that it was entirely possible to realize our “impossible dream” of reaching the many, surmounting the huge barriers of public ignorance and ultimately changing societal attitudes and removing stigma from alcoholism. … This was the Honorable Harold Hughes, recovered alcoholic, former governor of Iowa, then a freshman U.S. Senator (Pike 1979, p. 237).

Immediately upon arriving in office, Senator Hughes was determined to move quickly in developing legislation addressing alcohol problems. To this end, he elected to take on the chairmanship in 1969 of a newly formed Special Subcommittee on Alcoholism and Narcotics of the Senate Labor and Public Welfare Committee, even though funds were not available for its operation. Instead, Senator Hughes found a growing body of volunteers to do the work and donated his fees from speaking engagements to provide the necessary funding to bring the issues of alcohol abuse and alcoholism before both Congress and the American people.

The first hearing of the Special Subcommittee on Alcoholism and Narcotics was held in Washington, DC, on July 30, 1969. Among those testifying at this event were Mann and Bill Wilson, one of the AA founders. According to Pike:

Bill Wilson’s testimony before the Senate Alcoholism Subcommittee was historic and it was electrifying. The members of the Subcommittee listened to him with respect and rapt attention as Bill sketched the history of AA, described alcoholism as only he could, spoke of the desperate need for research and made an impassioned plea for long overdue and desperately needed Federal legislation and funding (Pike 1979, p. 240).

In 14 hearings held across the country during the summer of 1969, the Special Subcommittee received testimony from scientists, religious leaders, politicians, alcoholism treatment providers, and recovered alcoholics—individuals of disparate backgrounds who came together to tell the Nation that it was time to do something about the problems of alcohol abuse and alcoholism.

Based on these hearings, on May 14, 1970, Senator Hughes introduced into the Senate S. 3835, a bill intended to provide a comprehensive Federal program that would address the prevention and treatment of alcohol abuse and alcoholism. At this point, the bill faced a long road to enactment. Not only did it need to pass both congressional houses, but it also had to be signed by President Nixon, whose Executive Branch opposed the creation of the proposed NIAAA.

Public testimony on S. 3835 was held in the Senate on May 21 and 25, 1970. Among those who testified were Peter Domick, U.S. Senator from Colorado; Luther A. Cloud, president of the National Council on Alcoholism; Maxwell Weisman, director of alcohol programs for the State of Maryland; Marvin A. Block, of the Committee on Alcohol and Drug Dependence of the American Medical Association; Morris E. Chafetz, of Massachusetts General Hospital (and later the first director of NIAAA); and Selden E. Bacon. The bill was passed unanimously by the Senate on August 10.

### House Approval

Despite this auspicious beginning, the timing of S. 3835’s passage by the Senate late in the second session of the 91st Congress made final enactment even more uncertain. As the bill reached the House floor, a crowded December calendar threatened to postpone its passage. If the House did not reach a decision by the end of its 1970 session, S. 3835 would have to begin the legislative process over again in the coming year. However, with the behind-the-scenes participation of a key Congressman, Pike managed to slip the bill into position for a vote “in the nick of time” (Pike 1979). A version of S. 3835 passed the House on December 15, placing the proposed NIAAA within NIMH instead of granting it independent status. Senator Hughes accepted the House version in the interest of time. S. 3835 had only one more hurdle before reaching enactment.

### Enactment

Once more poised on the brink of success, those in the alcohol field were dismayed to learn that the new P.L. 91–616 might not become a “law of the land.” According to Pike, members of President Nixon’s cabinet had advised him to veto the bill. Pike, along with other influential participants in the movement to pass the legislation,^3^ joined “in a concerted effort to persuade the President to sign this legislation into law.” On New Year’s Eve in 1970, President Nixon signed P.L. 91–616, which Pike called “a landmark in Public Health Legislation …[that] came to be known as alcoholism’s Magna Carta” (Pike 1979, p. 241).^4^ There was no public ceremony. Very few people outside the nascent alcohol field were aware that history was being made. Yet December 31, 1970, marked not a year’s ending but an Institute’s beginning.

## P.L. 91–616: The Creation of NIAAA

The landmark legislation that created NIAAA represented to many in the alcohol field a point of culmination in the history of Americans’ answers to the alcohol problem. In his foreword to the *First Special Report to the U.S. Congress on Alcohol and Health* issued in December 1971, Secretary of Health, Education, and Welfare (HEW) Elliot L. Richardson noted:

We have emerged from an era when alcohol abuse and alcoholism were equated by the public with moral degeneration and despair to the day in 1970 when President Nixon signed into law the landmark Public Law 91–616. … This law followed a historical precedent of bringing together diverse and often divided interests in our society in support of a major public health measure (NIAAA 1973, p. V).

### NIAAA’s Mission

P.L. 91–616 established NIAAA as an organizational component of NIMH and instructed NIAAA to

… develop and conduct comprehensive health, education, research, and planning programs for the prevention and treatment of alcohol abuse and alcoholism and for the rehabilitation of alcohol abusers and alcoholics (P.L. 91–616, p. 1).

In addition to creating NIAAA, P.L. 91–616 did the following:

- Required that alcoholism programs be made available to Federal civilian employees
- Authorized the appropriation of Federal funds to the States via a formula grant mechanism to assist them in planning, establishing, maintaining, coordinating, and evaluating projects for the development of more effective prevention, treatment, and rehabilitation programs
- Prohibited discrimination in the hiring and firing of recovered alcoholics in nonsecurity jobs
- Authorized grants and contracts for education and training purposes and for demonstration and evaluation projects that provide treatment and prevention services
- Required the admission of alcohol abusers and alcoholics to any public or private general hospital receiving Federal funds for alcoholism treatment programs on the basis of medical need, and prohibited discrimination against this population solely because of their alcoholism
- Required that the records of patients in alcoholism treatment be kept confidential
- Established a National Advisory Council on Alcohol Abuse and Alcoholism to advise, consult with, and make recommendations to the Secretary of HEW on matters relating to the activities and functions of the Secretary in the field of alcohol abuse and alcoholism.

Interestingly enough, particularly in light of NIAAA’s present research mission and the testimony prior to 1970 by many in the alcohol field of the need for scientifically based knowledge about alcohol abuse, alcoholism, and related problems, the original law establishing NIAAA did not include a specific section relating to research. This function, rather, was authorized through the broad research authorities of Section 301 of the Public Health Service Act. It was not until the passage of P.L. 94–371 in 1976 that NIAAA gained a discrete research authority.

## Independence

**Figure 1:**
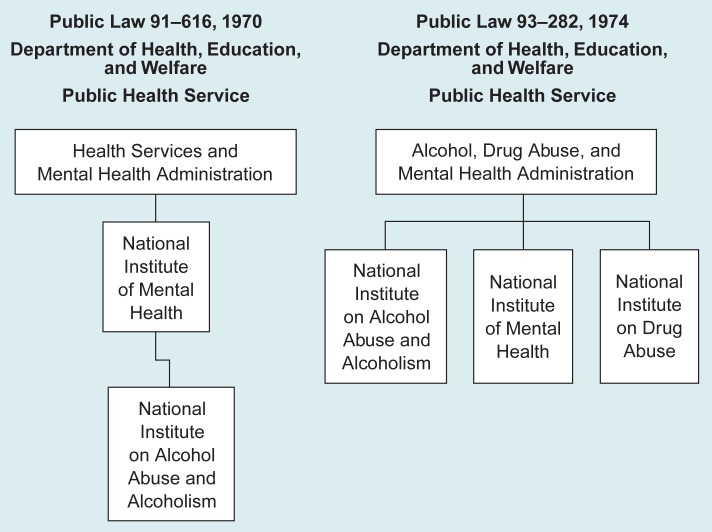
The National Institute on Alcohol Abuse and Alcoholism’s (NIAAA’s) placement within the Department of Health, Education, and Welfare’s Public Health Service. In 1974 NIAAA became an Institute independent from the National Institute of Mental Health.

Members of the alcohol movement who felt strongly that the needs of alcoholic persons would not receive the national attention and priority necessary to effect change if NIAAA remained under the mental health mantle continued to push for NIAAA’s independent status. Even before P.L. 91–616 was enacted, many organizations supporting the law had favored placing NIAAA on its own within HEW’s U.S. Public Health Service (Lewis 1988). Although Senator Hughes had accepted the House’s placement of NIAAA within NIMH in 1970, NIAAA’s autonomy had been his original intention in S. 3835. Thus, the stage was set for yet another major event in NIAAA’s history—its elevation from an Institute of NIMH to a fully autonomous Institute of the newly created Alcohol, Drug Abuse, and Mental Health Administration (ADAMHA; figure 1).

This reorganization occurred when NIAAA’s 3-year authorization went before Congress for renewal in 1973 and resulted in P.L. 93–282, the Comprehensive Alcohol Abuse and Alcoholism Prevention, Treatment, and Rehabilitation Act Amendments of 1974. These Amendments placed NIAAA, NIMH, and a new National Institute on Drug Abuse as equal partners under ADAMHA (figure 1).

In his preface to the proceedings of NIAAA’s Third Annual Alcoholism Conference, then-NIAAA director Morris Chafetz noted that “This time the signing took place—not in the dark stillness of the night—but in the bright ceremonial atmosphere of the Oval Office” (Chafetz 1974, p. iii). To many, the creation of a separate, visible national Institute devoted to addressing comprehensively the problems of alcohol abuse and alcoholism was the true ending of the story that had begun some 35 years ago.

## Epilogue

Since the passage 25 years ago of P.L. 91–616, NIAAA’s mission has evolved significantly. Changes have occurred in its leadership, its organizational home, and its primary program emphasis. True to the legacy of P.L. 91–616, however, NIAAA’s commitment to discovering the best ways to prevent and treat alcohol abuse and alcoholism in America still embodies the strong hopes and dedication of those who fought for this Institute’s creation and who helped to define alcohol abuse and alcoholism not as problems experienced by a few but as a national problem.

## Footnotes

1 The early temperance movement advocated “temperate,” or moderate, use of alcohol. This began to change to an antialcohol message around the 1850’s.

2 The Cooperative Commission on the Study of Alcoholism was established by a grant from the National Institute of Mental Health in 1961. It was the first national body established by the Federal Government to assess what the country was doing to respond to alcohol problems and to recommend ways to improve existing alcohol-related policies and programs.

3 Members of the movement who joined Pike in persuading the President included James Kemper, of the Kemper Insurance Company; Brinkley Smithers, of the Smithers Foundation; and Donald Kendall, of Pepsi Cola.

4 **Author’s Note:** In reading the personal accounts of the late Thomas Pike, who participated in the Special Subcommittee hearings, and the record of those hearings, this is the only reference I have seen to P.L. 91–616 as the alcoholic’s “Magna Carta.” Most of the witnesses who testified before the Special Senate Subcommittee referred to P.L. 91–616 as the “Alcoholics’ Bill of Rights.” Given Pike’s deep and abiding belief that alcoholics can be set free from their dependency, perhaps the use of Magna Carta as the quintessential basis for all human rights is the more appropriate analogy.
